# A Robust, High-Titer, Semi-Automated, and In-Culture Antibody-Capturing Transient CHO Platform Technology

**DOI:** 10.3390/antib14040087

**Published:** 2025-10-11

**Authors:** Lauren Gebhardt, Molica Abel, Jing Zhou, Audrey M. Vogt, Bo Hee Shin, Sarah L. Herrick Wagman, Ana Santos, Jerome Puginier, Florian M. Wurm, Maria J. Wurm, Guoying Grace Yan, Adedolapo Adeniyi, Sean K. H. Lim, Will Somers, Laura Lin, Aaron M. D’Antona, Xiaotian Zhong

**Affiliations:** 1 BioMedicine Design, Preclinical and Translational Sciences, Pfizer Research and Development, 610 MainStreet, Cambridge, MA 02139, USA; lauren.gebhardt@pfizer.com (L.G.); adedolapo.adeniyi@pfizer.com (A.A.); aaron.dantona@pfizer.com (A.M.D.); 2 MagellanBiologics, Parque Tecnológico de Cantanhede Edificio III Laboratório 5, 3060-197 Cantanhede, Portugal; 3 Life Science Faculty, Swiss Federal Institute of Technology Lausanne, 1015 Lausanne, Switzerland; 4 ExcellGene SA, Route d’ile au Bois 1a, 1870 Monthey, Switzerland

**Keywords:** transient gene expression, in-culture antibody capturing, CHO4Tx^®^, magnetic ProA beads, semi-automation, GenScript AmMag™ SA-Plus, AKTA Pure^TM^

## Abstract

Background: Recent advances in antibody discovery technologies, especially progress in de novo synthesis through machine learning, have imposed a significant production challenge for the generation of a large diversity of antibodies against nearly any target of interest. There is a demand for the rapid production of dozens of purified antibodies in 10-milligram quantities sufficient for functional screening and molecular assessment studies. Objectives: To meet this requirement, a semi-automated production methodology and workflow was developed to bridge the miniaturized high-throughput screenings (HTSs) and the conventional custom-scale workflow by taking advantage of four new technology applications. Methods: First, it exploited a novel, simple, high-titer transient expression system, “CHO4Tx^®^”, which could achieve high yields in the range of 200 mg/L and above, across a variety of antibody constructs, including challenging targets. The consistently high yields from this transient CHO platform enabled the delivery of ~20 mg of crude material per 100 mL scale flask production with a throughput capacity of nineteen constructs in a single run. Secondly, we established a magnetic ProA bead in-culture antibody-capturing process, which significantly shortened the production timeline by eliminating the steps of cell centrifugation, filtration, and medium column loading. Third, we utilized the GenScript AmMag™ SA Plus semi-automation, which could handle magnetic ProA bead elution for 12 constructs within less than 1 h. Lastly, we transformed the AKTA Pure^TM^ system into an automated buffer exchange purification system with a capacity of processing 19 samples in a single run. Results and Conclusions: This new production platform was proven to be robust and could be applied for the routine production of antibodies of sufficient quality and quantity in support of cell-based assays and biophysical characterization.

## 1. Introduction

Advancements in protein engineering driven by high-quality display technologies, immunization, and single-cell isolation [1,2,3,4] have generated many potential antigen binders. Most recently, “lab in the loop approaches”, such as de novo protein synthesis, utilization of protein language models, computational design, and deep learning [5,6,7,8,9], have emerged as promising new methods for antibody design and optimization. The traditional discovery technologies and the in silico approaches both require efficient hit evaluations. This has, therefore, created an urgent need for rapidly generating thousands of isolated and engineered antibody proteins with an unknown expression performance via high-throughput screening (HTS) methods, as well as a demand for a mid-scale production process for a sizable number of follow-on molecules.

Transient gene expression (TGE) technologies, which can produce moderate amounts of proteins for many molecules, have been utilized to expedite discovery testing cycles. The TGE technologies exploit episomal plasmid DNAs for protein synthesis in mammalian cells without chromosomal integrations [10,11,12,13] and can, therefore, provide protein in days for screening assays, functional investigations, and preclinical analysis during discovery and early development. They have been routinely utilized for antibody optimization and engineering for discovery acceleration, as multiple rounds of engineering require a fast generation of various antibody constructs.

Both human embryonic kidney (HEK)-293 cells and Chinese hamster ovary (CHO) cells are major cell hosts for the TGE systems. HEK293 exhibits high transfectability and reproducibility at high cell densities under serum-free suspension culture conditions, yet HEK293 cells are not commonly used clinically [14]. They can also generate unwanted post-translational modifications different from those produced in CHO cells [15,16,17,18,19], which can theoretically affect protein folding and pharmacokinetic properties. Therapeutic proteins derived from HEK293 cell culture could also possess potential risks of inadvertently transferring human viruses, including ancient, reactivated retroviruses, from thousands of endogenous retroviral sequences to patients [14,20]. Compared with transient expression in HEK293 cells, CHO cells initially attained modest transient expression yields [21,22]. Yet over the past decade, the transient CHO system has achieved significantly higher productivities [23,24,25,26,27,28,29,30]. Owing to the host consistency with downstream manufacturing processes, TGE technologies in CHO cells possess a unique advantage of uncovering potential issues for candidate molecules at the early stages of drug development.

The increasing speed in antibody discovery has also driven purification development with automation and high capacity of throughput [31,32,33,34]. Miniaturized high-throughput (HTP) plate-based or tube-based purification has been widely adopted for material generation to meet the small protein requirement for relatively simple screening. However, this HTP method cannot provide enough antibodies with suitable purity for more sophisticated assays. Biophysical characterization and in vitro functional testing require substantially larger quantities of purified antibodies of at least 10 milligrams (mg). High-throughput purification (up to 240 samples) with the incorporation of an autosampler from 0.5 to 15 mL of culture volume [35] and a two-step preparative purification with an autosampler from 35 mL of clarified cell supernatants with yields of 1–5 mg [36] have been reported.

Typical antibody production procedures involve clarification of cells from cell culture via centrifugation and/or filtration, as well as ProA column loading of conditioned media for protein capturing. These steps are laborious and time-consuming, requiring equipment and materials. The cell separation step can potentially increase host cell protein (HCP) levels and proteolytic activity in the clarified materials. To address these limitations, different production procedures for cell clarification and antibody capturing need to be developed and utilized.

To meet the demand for rapid production on the 10 mg scale, we have developed a semi-automated novel production methodology and workflow by taking advantage of a high-titer transient CHO expression system for 100 mL scale productions. A magnetic ProA bead in-culture antibody-capturing process for transiently transfected CHO cells, coupled with the utilization of the GenScript AmMag™ SA Plus semi-automation system, eliminated the steps of cell clarification, filtration, and medium loading in a throughput manner. An AKTA Pure^TM^ system was converted into an automated buffer exchange purification system capable of processing 19 samples in a single run. This newly established production process could support the weekly generation of dozens of antibody proteins with adequate quality and quantity for drug discovery characterizations.

## 2. Materials and Methods

### 2.1. Cell Culture and Transient Transfection

For the CHO4Tx^®^ cells (MagellanBiologics, Cantanhede, Portugal), 4 × 10^7^ cells were thawed by hand or by incubation in a 37 °C water bath. These cells were dispensed rapidly into 7.0 mL of pre-warmed CHO 4Tx^®^ Cultivation Medium (CM, MagellanBiologics) containing 4 mM of L-glutamine (L-Gln) (Thermo Fisher Scientific, Waltham, MA, USA). The cells were pelleted via centrifugation at 3000× *g* for 5 min (min) and re-suspended in 25 mL of pre-warmed fresh CHO 4Tx^®^ CM in a 125 mL Erlenmeyer Shake Flask (Corning Life Sciences, New York, NY, USA). The cells were incubated at 110 rpm, 37 °C, and 8% CO_2_. Viable cell density (VCD) and percentages of cell viability were evaluated via a ViCell BLU cell viability analyzer (Beckman Coulter, Indianapolis, IN, USA) to confirm an acceptable range of >95% cell viability and a 2.0 × 10^6^ cells/mL VCD. Within two or three days, cells proliferated to the target density of 4.0–6.0 × 10^6^ cells/mL, with >97% viability, and were passaged on day 2 to the target density of 0.7 × 10^6^ cells/mL or passaged on day 3 to the target density of 0.3 × 10^6^ cells/mL in 100 mL of pre-warmed CHO 4Tx^®^ CM. For Expi293F™ cells and ExpiCHO-S™ cells (Thermo Fisher Scientific), procedures for cell maintenance and transient transfection were followed as previously described [30].

### 2.2. DNA Complexation Using the Tecan Fluent Automation System

A custom script was developed using the Tecan FluentControl software version 2.7 (Mannedorf, Switzerland) to automate DNA complexation for TGE workflows. The script was integrated with GeneData Biologics (Basel, Switzerland) to retrieve experimental parameters, appropriate plasmid DNA combinations were generated, and a worklist was exported. The worklist was then imported into FluentControl to instruct the Tecan Fluent-480 liquid handler on the precise volumes and combinations of DNA required. Plasmid mixtures were calculated based on the target cell line and its DNA concentration requirements (6.5 mg/L for CHO4Tx^®^, and 1 mg/L for Expi293F™ or ExpiCHO-S™). The volume of each vector contributing to the plasmid combinations was determined according to the concentration and identity of the specific vector batches used. Barcode labeling was employed to ensure traceability and correct vector selection.

### 2.3. Transient CHO4Tx^®^ Production at 500 mL Scale

One day before transfection, VCD and cell viability were evaluated to confirm an acceptable cell density range of 4.0–6.0 × 10^6^ cells/mL and a viability of >98%. Cells were aliquoted to 2.0–3.0 × 10^6^ cells/mL and pelleted via centrifugation at 3000× *g* for 5 min (min). The cell pellets were resuspended in 500 mL of pre-warmed CHO 4Tx^®^ CM in a 2 L Corning flask (Corning, Glendale, AZ, USA) and incubated at 37 °C with an orbital shaking speed of 140 rpm in a Kuhner shaker incubator with an orbital throw of 25 mm and 8% CO_2_. On the day of transfection, the VCD and cell viability were evaluated to confirm the acceptable cell density range of 4.0–6.0 × 10^6^ cells/mL (VCD) and a viability of >98%. The cells were pelleted via centrifugation at 3000× *g* for 10 min and resuspended in 250 mL of pre-warmed CHO 4Tx^®^ Transfection Medium (TM, MagellanBiologics) in a 3 L Corning flask to achieve the target cell density of 1.2 × 10^7^ cells/mL. Plasmid DNAs were prepared as a total of 3.25 mg of DNA consisting of light chains (LCs) and heavy chains (HCs) in the mass ratio of 1:1. The DNAs were added to a 3 L Corning flask containing 250 mL of CHO 4Tx^®^ cells suspended in CHO 4Tx^®^ TM. These cells with the DNAs were incubated for 3 h at 31 °C, with a shaking speed of 120 rpm and 5% CO_2_. An amount of 250 mL of pre-warmed CHO 4Tx^®^ production medium (PM, MagellanBiologics) was added to achieve a final TGE volume of 500 mL. The transfected culture flasks were transferred into Kuhner shaker incubators (Kuhner AG, Basel, Switzerland) or Multitron shaker incubators (Infors HT, New York, NY, USA), set at at 31 °C, 120 rpm, and 5% CO_2_ for up to 14 days.

### 2.4. Transient Transfection of CHO4Tx^®^ Cells at 100 mL Scale

One day prior to the transfections (−1 day), the CHO4Tx^®^ cell viability and VCD should have been >95.0% and between 4 and 6 × $10^{6}$ cells/mL, respectively. The cells were pelleted via centrifugation at 3000× *g* for 4 min and then resuspended in 100 mL of prewarmed CHO4Tx^®^ CM to achieve a density of 2.3 × $10^{6}$ cells/mL in either 500 mL Corning flasks or 250 mL Thomson flasks (Thomson, Carlsbad, CA, USA). The cells were incubated at 37 °C, 8% CO_2_, and 100 rpm overnight. On the transfection day (day 0), the CHO4Tx^®^ cell viability and VCD should have been >95% and around 6 × $10^{6}$ cells/mL, respectively. The cells were pelleted at 3000× *g* for 5 min and resuspended in 50 mL of pre-warmed CHO4Tx^®^ Transfection Medium in 250 mL Thomson flasks to achieve a density of 1.2 × $10^{7}$ cells/mL. After resuspension, 0.65 total mg of DNA (0.325 mg of HCs and 0.325 mg of LCs) were added to each flask. The culture was then incubated at 31 °C and 5% CO_2_ with a shaking speed of 140 rpm for 3 h. After 3 h, 50 mL of pre-warmed CHO4Tx^®^ Production Medium was added to the flask to achieve a final volume of 100 mL and a VCD of 6.0 × $10^{6}$ cells/mL. The transfected cultures were maintained at 31 °C, 5% CO_2_, and 140 rpm in Corning flasks or at 160 rpm in Thomson flasks for 14 days.

### 2.5. Post-Transfection Data Acquisition and Determination of mAb Concentration

At various time points post-transfection (d3, d5, d7, d10, d12, and d14), 2 mL of the culture medium was collected from each construct. An amount of 1 mL was used to measure viability and VCD with the Vi-CellXR. The other 1 mL was used to measure titer in duplicate with the Octet RED96e system (Sartorius, Göttingen, Germany). A standard curve was generated for the Octet using serial dilutions of a control IgG in Dulbecco’s phosphate-buffered saline (PBS). The standard curve generated for day 3 was saved and applied to the samples throughout the time course.

### 2.6. Evaluating Effects of Incubation Time and Elution Buffers on Control IgG In-Culture Capturing with Magnetic ProA Beads

A magnetic bead slurry of 50% AmMag™ ProA magnetic beads (GenScript, Piscataway, NJ, USA) to 50% 20-ethanol was prepared. An amount of 100 mL of non-transfected wild-type CHO4Tx^®^ cells at a cell density of ~6 × $10^{6}$ cells/mL cultured in CHO4Tx^®^ culture medium was spiked with 5 mg of a purified control, IgG. An amount of 2 mL of magnetic bead slurry was spiked into each culture. The cultures were incubated at 31 °C, 5% CO_2_, and 140 rpm for various durations of time (15 min, 30 min, 1 h, 2 h, 3 h, 4 h, and 24 h). After the designated incubation periods, each culture was divided into two 50 mL conical tubes. Each tube was placed on the GenScript AmMag™ MR magnetic separation rack. The cell supernatant was poured off, leaving behind only the magnetic beads with bound protein. An amount of 10 mL of PBS was added to each conical tube and moved to a tube revolver (Thermo Fisher Scientific) to perform end-to-end (bottom-to-head of conical tubes) rotation for 5 min. After 5 min, the PBS was poured off, and an additional 10 mL of PBS was added to each tube for a second wash round. After the second washing, the PBS was poured off, and 2 mL of either a pH 3.0 or pH 3.5 elution buffer (150 mM glycine and 40 mM sodium chloride) was added to one of the conical tubes for each incubation duration. The tubes were placed back on the rotator for 10 min. After 10 min, the tubes were placed on the magnetic separation rack, and the elution buffer was collected and neutralized with 200 µL of 2M 4-(2-hydroxyethyl)-1-piperazineethanesulfonic acid (HEPES) (pH 8.0). The IgG concentration of each eluate was measured with Nanodrop Microvolume Spectrophotometers (ThermoFisher Scientific).

### 2.7. Semi-Automatic Purification of mAb Using SA Plus and Magnetic ProA Beads

After a 14-day CHO4Tx^®^ transfection duration, 2 mL of magnetic bead slurry [50% (*v*/*v*) beads in 20% ethanol] was added directly to the culture flask. The flask was transferred back to the incubator to incubate with the magnetic beads for 30 min. After the incubation period, the entire contents of the flask were transferred into a single 50 mL conical tube using the GenScript AmMag™ MR magnetic rack (GenScript). A 50 mL conical tube was placed in the magnetic separation rack, and 50 mL of the cell culture with the beads was poured into the tube. After a few seconds, the ProA magnetic beads were pelleted, and the cell culture was poured off. Then, the remaining 50 mL of the culture with the beads was poured into the same 50 mL conical tube. The conical tube was then placed in the SA Plus Semi-Automated System (GenScript). The machine was programmed to perform 2 washing rounds with PBS, followed by 2 elution rounds with pH 3 elution buffer. After each elution round, the machine paused for manual collection of the eluate. When all rounds of elution were collected, they were neutralized in 2M HEPES (pH 8.0), and the protein concentration was measured using a Nanodrop.

### 2.8. CHO Host Cell Protein (HCP) Content Measurement

A CHO HCP ELISA was performed on all samples using the CHO 3rd generation ELISA kit (Cat#F550-1, Cygnus Technologies, Leland, NC, USA) according to the manufacturer’s protocol [https://www.cygnustechnologies.com/media/productattach/import/F550%20PI_CHO%203G%20ELISA_Rev%204.pdf (Assessed on 10 October 2025)]. The standard curve was plotted in duplicate using the standards included in the kit. All ProA samples were normalized to an IgG concentration of 0.1 mg/mL and plated in duplicate. Positive controls were created for each ProA sample and plated in duplicate by spiking 12.5 ng of standard F into each sample. The HCP concentration was calculated by reading the absorbance at 450/650 nm on SpectraMax M3 (Molecular Devices, San Jose, CA, USA) and plotting the OD readout from samples against the standard curve on the SoftMax Pro 7.0.3 software.

### 2.9. Sephadex G-25 Column Chromatography

The ÄKTA Pure 150 (Cytiva, Marlborough, MA, USA) chromatography system was adapted with Inlet Valve V9H-X1 and V9H-X2 (Cytiva), tethered together and to the S7 position on the Sample Inlet Valve Kit V9H-IS (Cytiva). An amount of 53 mL of HiPrep desalting columns with Sephadex G-25 resin (Cytiva) was equilibrated with 2 column volumes (CVs) of equilibration buffer. An amount of 1 to 4 mL of ProA affinity-purified antibody protein at 0.5–12.5 mg/mL was loaded onto the G-25 desalting column equilibrated in PBS (pH 7.2). Isocratic protein elution in PBS was applied for 3CV, and fractions were collected containing purified protein. In-line and column clean-in-place (CIP) were performed simultaneously with sodium hydroxide and the equilibration buffer position on the V9H-X2 (Cytiva), ensuring 15–30 min of contact across the system-shared sample lines, column, and OUTLET fraction collector. The same flow path was flushed and neutralized using the equilibration buffer position on the V9H-X2 (Cytiva).

### 2.10. Statistical Analysis

The variance reported was reflective of the observed mean ± standard error of the mean of two independent experiments. *p*-values were calculated with two-way ANOVA statistical analysis, performed using the GraphPad Prism version 9 software (GraphPad Software, San Diego, CA, USA) with the pairwise R function *t*-test.

## 3. Results

### 3.1. The CHO4Tx^®^ System Is a High-Titer Transient CHO Expression System

CHO4Tx^®^ cells established by MagellanBiologics were derived as a non-cloned cell population from CHOExpress^®^ cells (ExcellGene SA, Monthey, Switzerland) originally adapted from serum-containing, adherent cultures to a suspension culture in an animal component-free (ACF) and chemically defined (CD) medium. After hundreds of subcultivations executed under high-growth-rate conditions (log phase and avoidance of lag phases) [37], these cells achieved a high cell density in batch culture (max. cell density >30 million cells/mL), with an absence of any aggregation. The density of 30 million cells/mL could be reached within 4 days at 37 °C, with a rich medium that did not require addition of glucose. They also had high transfectability and viability for up to 14 days after transfections, maintaining cell robustness under high shear forces. The average doubling time for CHO4Tx^®^ was ~18.5 h.

Avoiding DNA and transfection reagent complex formation, as well as eliminating extra feed during the protein production phase, CHO4Tx^®^ provides a simplified TGE process [38]. The proprietary CHO4Tx^®^ transfection medium contains several ingredients for interaction with nucleic acids. The components considered critical in DNA delivery to cells were charged molecules and ions that contributed to particle formation, including metal ions, and positively charged polymers. A composition was developed to minimize cell toxicities in transfections. There were likely other components in the 60+ chemistries of the transfection medium that played a part in generation of DNA complexes that interact with the cells. These were not determined.

**Figure 1:**
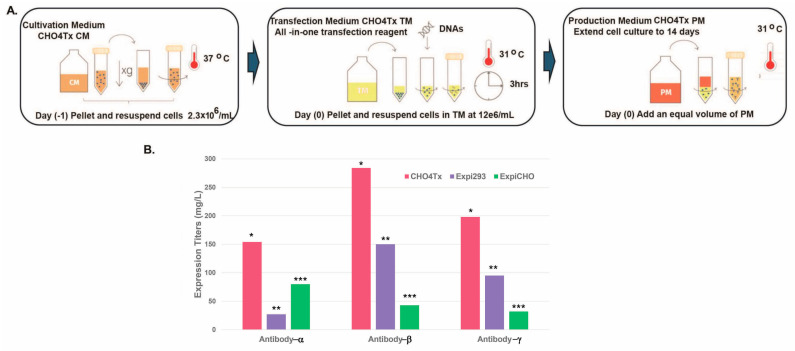
CHO4Tx^®^ is a high-titer transient CHO expression system compared with transient Expi293F™ and ExpiCHO-S™ for several low-expressing antibodies. (**A**) CHO4Tx^®^ transfection workflow. (**B**) Expression comparison between CHO4Tx^®^, Expi293F™, and ExpiCHO-S™. Transfections in CHO4Tx^®^, Expi293F™, and ExpiCHO-S™ on the scale of 500 mL were performed as described in the Materials and Methods. * vs. **, *p* = 0.081; * vs. ***, *p* < 0.05; * vs. ** and ***, *p* < 0.05.

As shown in Figure 1A, the TGE process in CHO 4Tx^®^ consisted of three media: a cultivation medium, a transfection medium, and a production medium. For the transfection process, the CHO4Tx^®^ cells, routinely maintained in the cultivation medium, were seeded one day before transfection (day 1). On the subsequent day (D0), these cells were resuspended in the transfection medium, which came complete with DNA transfection reagents included. Plasmid DNAs encoding antibody proteins were inoculated directly into the cell culture at 31 °C. After culturing for 3 h, the production medium was added for extended culturing for up to 14 days. This three-step protocol leads to a high yield, with excellent reproducibility between transfections.

To further evaluate this new system, we selected three difficult-to-express antibody targets that showed low titers in either transient Expi293F™ or transient ExpiCHO-S™ systems. As shown in Figure 1B, these three low expressors were simultaneously produced for a direct transient production comparison between CHO4Tx^®^ and Expi293F™/ExpiCHO-S™ in a 0.5 L production volume. The CHO4Tx^®^ system was found to be effective in expressing all three low expressors and outperformed both Expi293F™ and ExpiCHO-S™ cells. For antibody-α, the CHO4Tx^®^ titer was 5.7-fold higher than that of Expi293F™ and 2-fold higher than that of ExpiCHO-S™. For antibody-β, the CHO4Tx^®^ titer was 2-fold higher than that of Expi293F™ and 6.6-fold higher than that of ExpiCHO-S™. For antibody-γ, the CHO4Tx^®^ titer was 2-fold higher than that of Expi293F™ and 6-fold higher than that of ExpiCHO-S™. The difference between CHO4Tx and ExpiCHO-S™ (*p* < 0.05) was significant. The difference between CHO4Tx and Expi293F™ (*p* = 0.081) was not significant, whereas the difference between CHO4Tx and Expi293F™/ExpiCHO-S™ combined (*p* < 0.05) was significant.

**Figure 2:**
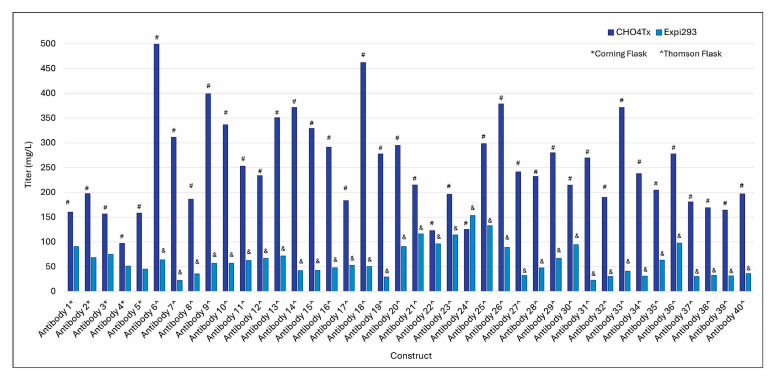
Production using 100 mL flask for CHO4Tx^®^ vs. Expi293F™. CHO4Tx^®^ and Expi293F™ transfections were performed at the scale of 100 mL in either a 500 mL Corning flask (*) or a 250 mL Thomson flask (ˆ), as described in the Materials and Methods. ^#^ vs. ^&^, *p* < 0.01.

To establish a workflow that could enable the delivery of ~10 mg of crude material, we developed a procedure for 100 mL culture volume production by adapting the protocol used for the 500 mL scale. As shown in Figure 2, most of these constructs achieved expression titers of around 200 mg/L and higher in CHO4Tx^®^, with some being >400 mg/L. The titers in CHO4Tx^®^ were, on average, five-fold higher than those in Expi293F™. The difference between CHO4Tx and Expi293F™ was significant (*p* < 0.01).

### 3.2. Production with 100 mL Flasks Could Be Further Optimized Through Cultivation in Different Vessels

**Figure 3:**
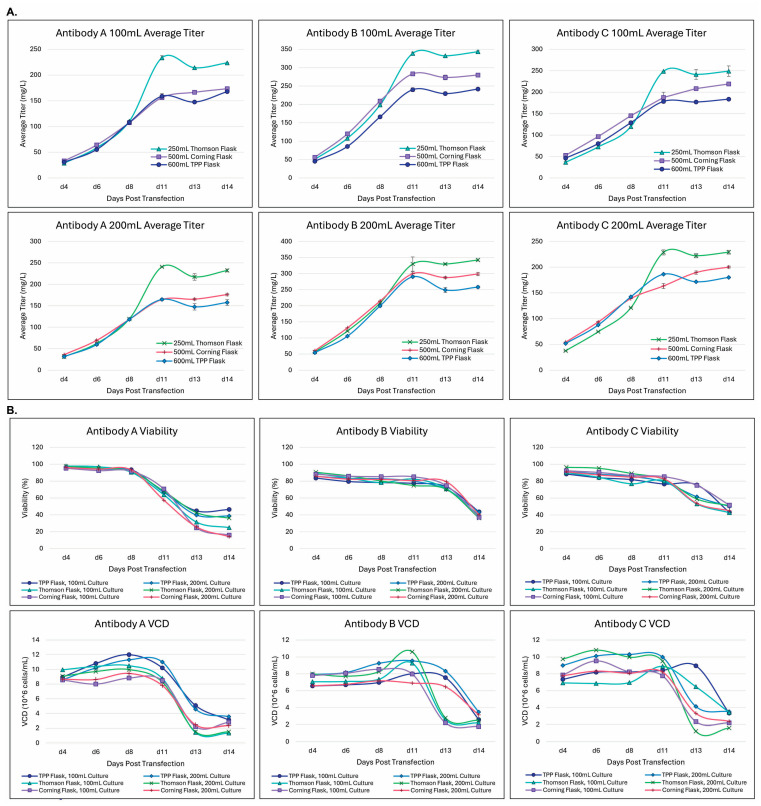
Production using 100 mL flasks was further optimized through Thomson flasks and 500 mL TPP bioreactors. CHO4Tx^®^ transfections were performed as described in the Materials and Methods. The shaking speeds for 500 mL and 1 L Corning flasks were 140 rpm, 250 rpm for TPP, and 160 rpm for 250 mL Thomson flasks and 500 mL Thomson flasks. All other conditions remained constant. (**A**) Expression titers (n = 2 ± SE). (**B**) Cell viability and VCD (n = 2 ± SE).

The 100 mL scale production was typically performed in 500 mL Corning^®^ Erlenmeyer flasks. It is known that culture vessels and appropriately chosen mixing principles by orbital shaking play a role in improving protein expression titers [39]. There are two additional types of flasks for cell culturing that are commercially available, i.e., Thomson Optimum Growth^®^ flasks and TubeSpin^®^ bioreactor 600 (TPP) [39]. We set out to compare the expression levels among these three different culturing flasks to select the best default vessel for the workflow. Three different antibodies were expressed simultaneously: antibody-A, antibody-B, and antibody-C. As shown in Figure 3A, three antibodies achieved high expression (>200 mg/L) in 100 mL culturing in these different flasks. Thomson flasks of 250 mL produced significantly higher titers than those of 500 mL Corning flasks and TPP 500 mL bottles for antibody-A (223 mg/L vs. 173 mg/L and 157 mg/L), antibody-B (343 mg/L vs. 279 mg/L and 241 mg/L), and antibody-C (249 mg/L vs. 219 mg/L and 183 mg/L). For 200 mL culturing, 500 mL Thomson flasks were still the best over 1 L Corning flasks or TPP 500 mL bottles (antibody-A: 232 mg/L vs. 176 mg/L and 157 mg/L; antibody-B: 342 mg/L vs. 298 mg/L and 257 mg/L; antibody-C: 229 mg/L vs. 200 mg/L and 180 mg/L). Figure 3B shows that the patterns for the cell viability and VCD up to day 11 across all transfections were similar. However, the day-13 VCDs for antibody-B and -C had significant differences, while those for antibody-A were similar among all transfections. Since the titer differences were not that dramatic among these transfections, the VCD variations at a later timeframe might not be a crucial factor. Based on these results, 250 mL Thomson flasks were, therefore, selected as the default vessel for the workflow.

### 3.3. Antibody Protein Could Be Captured Through In-Culture Magnetic ProA Bead Incubation

For antibody production procedures, there are critical, laborious, and time-consuming steps involving the clarification of cells from cell culture via centrifugation and/or filtration, as well as ProA column loading of conditioned media for protein capturing. To establish a robust workflow in conjunction with the transient CHO4Tx^®^ expression, we instituted a new production workflow by inoculating magnetic beads directly into the cell culture so that the steps of clarification, purification, and medium column loading could be skipped.

**Figure 4:**
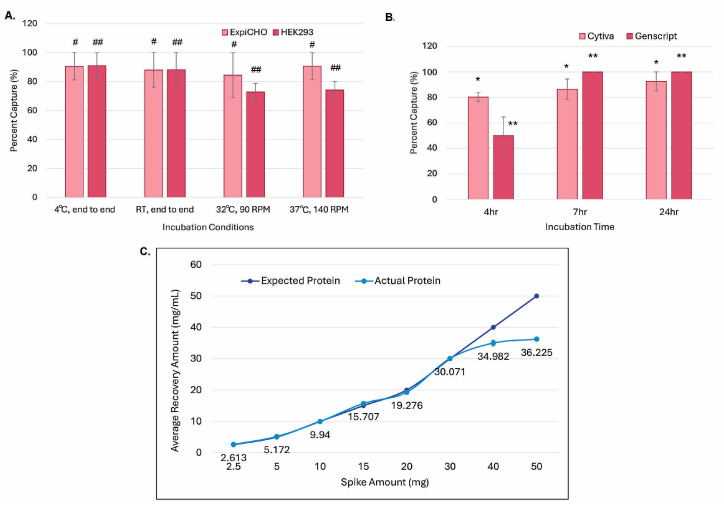
Antibody protein could be captured through in-culture magnetic bead incubation. Magnetic ProA bead in-culture binding and elution were performed as described in the Materials and Methods. (**A**) Antibody-capturing efficiency was compared between conditions with different temperatures, RPM values, and incubation times (n = 2 ± SE; “ ^#^ and ^##^”, *p* = 0.58; “^#^” vs. “^##^”, *p* = 0.32) in 100 mL of ExpiCHO-S™ cells at a cell density of ~6 × $10^{6}$ cells/mL at 32 °C at 90 rpm or in 100 mL of Expi293F™ cells at a cell density of ~3 × $10^{6}$ cells/mL at 37 °C at 140 rpm, with purified control IgG protein. (**B**) Antibody-capturing efficiency comparison between magnetic ProA beads from Cytiva (n = 2 ± SE) or GenScript (n = 2 ± SE) (“* and **”, *p* < 0.05; “*” vs. “**”, *p* = 0.73) in 100 mL of non-transfected CHO4Tx^®^ cells at a cell density of ~6 × $10^{6}$ cells/mL with purified control IgG protein at 140 rpm at 31 °C. (**C**) Determination for maximum antibody-binding capacity of magnetic ProA beads from GenScript (n = 2 ± SE) in 100 mL of non-transfected CHO4Tx^®^ cells at the cell density of ~6 × $10^{6}$ cells/mL with purified control IgG protein at 140 rpm and 31 °C.

As shown in Figure 4A, magnetic ProA beads were added to different cell cultures for different incubation times, temperatures, and rotational speeds, i.e., in CHO4Tx^®^ and ExpiCHO-S™ at 32 °C with 90 rpm and in Expi293F™at 37 °C at 140 rpm. The magnetic ProA beads were collected manually with magnetic racks, and the captured antibody proteins were eluted with the typical antibody elution buffer (150 mM glycine and 40 mM sodium chloride (pH 3.5)). As shown in Figure 4A, the capturing efficiencies over time for both cell lines across the culture conditions at 4 °C, room temperature (RT), 32 °C, and 37 °C were similar, with no significant differences (*p* = 0.58 among the four conditions, and *p* = 0.32 between the two cell hosts). Under this elution condition, antibody capturing with magnetic beads from Cytiva started to be saturated at 4 h, whereas that for beads from GenScript started at 7 h (Figure 4B). Elution efficiency differences among the 4 h incubation and the 7 h/24 h incubations were statistically significant (*p* < 0.05), whereas the efficiency differences between the Cytiva resin and the GenScript resin were not significant (*p* = 0.73). As shown in Figure 4C, the maximum binding capacity for GenScript magnetic ProA beads was determined to be ~36 mg/mL.

### 3.4. Decreasing Elution pH to 3.0 Dramatically Increased Elution Efficiency and Reduced In-Culture Capturing Time

**Figure 5:**
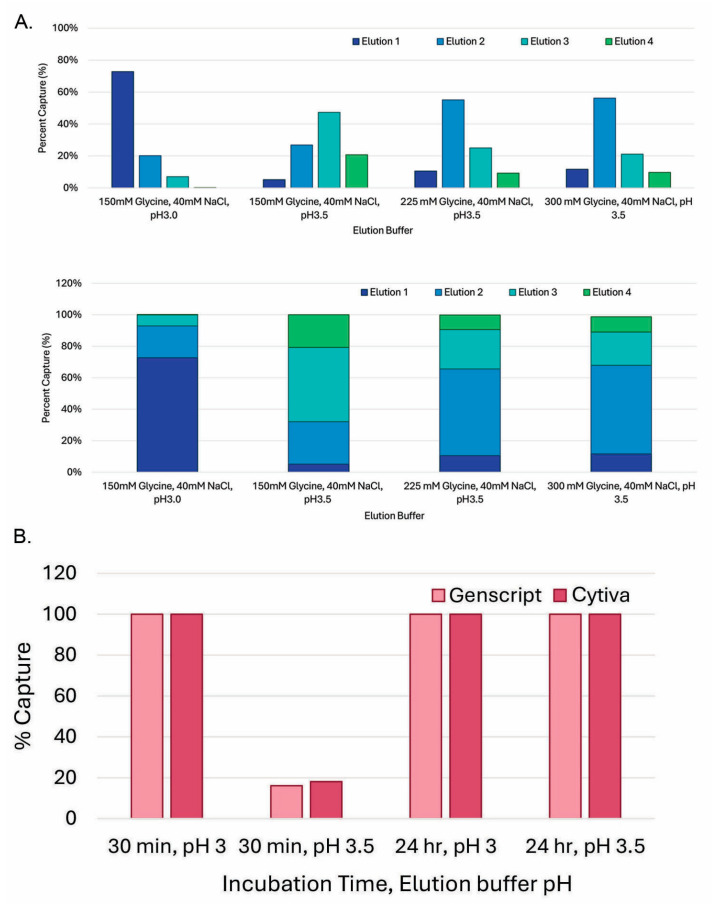
Decreasing elution pH to 3.0 dramatically increased elution efficiency and decreased in-culture capturing time. (**A**) Elution efficiency with different pH values and salt concentrations. ExpiCHO-S™ cultures of 100 mL were spiked with 5 mg of control IgG and 2 mL of GenScript 50% bead slurry. After 24 h of incubation at 32 °C and 90 rpm, magnetic bead elution was performed as described in the Materials and Methods with various elution buffers indicated. (**B**) Capturing efficiency comparison between magnetic ProA beads from Cytiva and GenScript during 30 min or 24 h of incubation.

To further optimize the magnetic bead-capturing processes, we triaged the elution steps with different pH values and salt concentrations. As shown in Figure 5A, at 150 mM glycine (pH 3.5), most of the antibody protein came out in the third round of elution (elution 3). When the glycine concentration was increased from 150 mM to 225 mM or 300 mM, most of the antibody protein came out in the second round of elution (elution 2), indicating that a stronger salt strength can, indeed, improve the elution efficiency of magnetic ProA beads. The most dramatic improvement was observed when the elution pH was decreased to pH 3.0, as we found that the pH of the magnetic ProA-beads with 150 mM glycine (pH 3.5) was around 3.8. Under the pH 3.0 condition, most of the protein came out in the first round of elution (elution 1). The pH for beads with 150 mM glycine (pH 3.0) was around 3.3. This is consistent with the hypothesis that the residual PBS buffer (pH 7.2) could contribute to the pH increase during the elution incubation. As shown in Figure 5B, the pH effects were similar between GenScript ProA beads and Cytiva ProA beads after the 30 min incubation and 24 h incubation.

**Figure 6:**
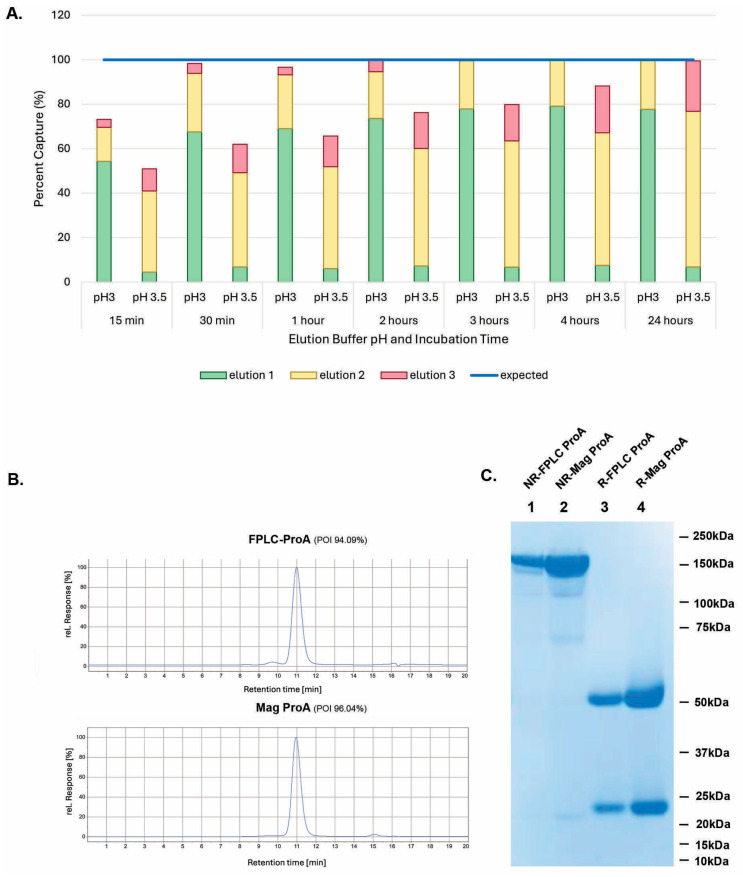
Magnetic ProA bead elution at pH 3.0. (**A**) Time course comparison between elution efficiencies of magnetic ProA beads in-culture capturing in CHO4Tx^®^ cell culture, eluted at pH 3.0 and 3.5. (**B**) Analytical SEC comparison between the protein samples purified by traditional FPLC column loading onto ProA resin (FPLC-ProA), eluted at pH 3.5, or by magnetic ProA bead in-culture capturing (Mag ProA), eluted at pH 3.0. (**C**) SDS-PAGE analysis of the protein samples under reduced (R) or non-reduced (NR) conditions.

Since pH in elution buffers was found to be critical to the purification efficiency for magnetic ProA beads, we repeated the experiments for the time course study with inoculation of magnetic ProA beads into cell cultures. As shown in Figure 6A, at pH 3.0, most of the protein was found in the first elution, whereas the highest protein concentration was found in the second elution when eluted at pH 3.5. Also, a 30 min incubation was enough for full antibody capturing, as most of the antibody protein was found in elutions 1 and 2 at pH 3.0, and very little was found in elution 3. In contrast, it took a 24 h incubation for a maximal elution at pH 3.5, and a substantial amount of the protein was still found in the third elution. Therefore, 30 min of incubation with magnetic beads and elution at pH 3.0 was selected as the default condition for the in-culture capturing workflow.

The product quality of magnetic ProA-purified samples (Mag-ProA) was compared with that of those purified by the conventional medium loading into ProA resin through FPLC (FPLC-ProA). As shown in Figure 6B, the POIs from the aSEC of both samples were comparable (96.04% vs. 94.09%). Figure 6C shows a similar purity result from SDS-PAGE. When CHO HCP ELISA was performed, the Mag-ProA-purified sample contained about 221 ng/mL of HCP in 1 mg/mL of IgG, whereas the HPLC-ProA-purified sample had about 311 ng/mL.

### 3.5. GenScript AmMag™ SA Plus System Enabled Automated Elution with 12 Constructs per Round in Under 1 h

**Figure 7:**
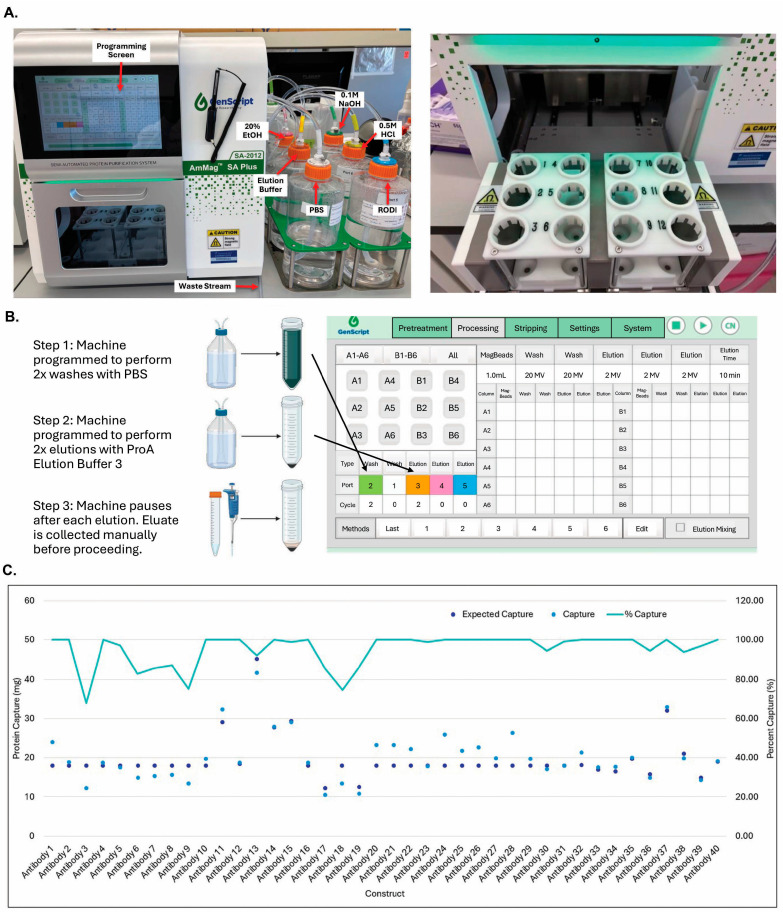
GenScript SA Plus system enabled automated elution with 12_constructs per round under 1 h. (**A**) Images of GenScript SA Plus system. (**B**) Programming steps for GenScript SA plus system. (**C**) Magnetic ProA capturing efficiency. Titers from 100 mL transient transfections in CHO4Tx^®^ were determined by HPLC-based ProA analysis. The protein produced for each of these constructs was captured using magnetic beads as described in the Materials and Methods. Protein capture was measured with the Nanodrop and compared with the titers obtained by the HPLC-based ProA analysis.

To increase the handling throughput, we evaluated automated systems in the market. The KingFisher Duo Prime System (ThermoFisher Scientific) can handle small volumes of magnetic bead purification, which can allow a larger scale of purification through repeated tubing. Most recently, GenScript launched an automated magnetic bead purification system for 50 mL volumes with 12 constructs per purification round (Figure 7A). The AmMag™ SA Plus machine can be programmed to perform two washes with PBS and two elutions with the pH 3.0 elution buffer. The machine pauses after each elution step and proceeds after the elution is collected (Figure 7B). As shown in Figure 7C, the purification efficiency with the SA Plus system was high (averaged ~95%), and the product qualities were comparable to those of traditional purification, as indicated by aSEC and SDS-PAGE.

### 3.6. Building V9HX1 and V9HX2 Values Facilitated Automated Buffer Exchange for 19 Constructs

**Figure 8:**
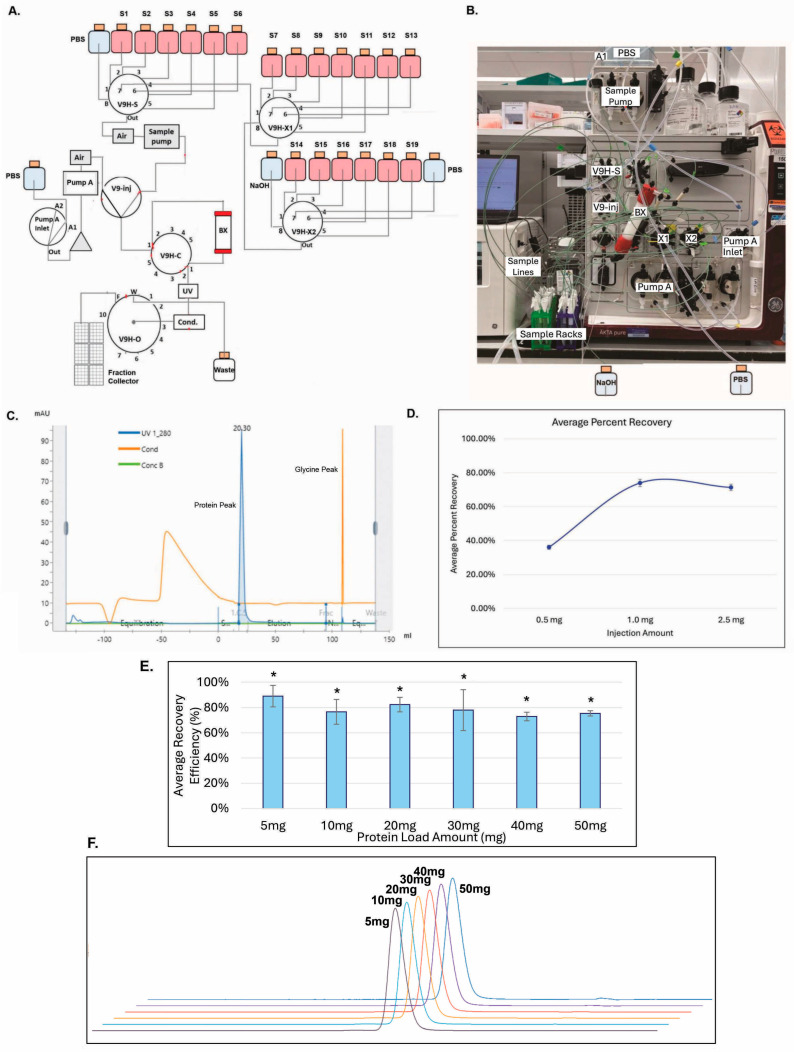
Building X1 and X2 values enabled automated buffer exchange with 19_constructs in 10 h. (**A**) Flow diagram for the AKTA Pure^TM^ X1 and X2 buffer exchange system. (**B**) The X1 and X2 AKTA Pure^TM^ system picture. (**C**) Chromatography of the buffer exchange step. (**D**) Calculated recovery efficiencies of the system (n = 19 ± SD). (**E**) Calculated recovery efficiencies for 5–50 mg antibody samples (n = 2 ± SE). (* *p* = 0.79). (**F**) Representative aSEC profiles for the buffer-exchanged samples generated in panel E.

To remove glycine and formulate the purified samples into proper buffers, a buffer exchange step typically needs to be in place during purification. We established a new workflow with AKTA Pure^TM^ by installing two additional versatile valves (V9H-X1 and V9H-X2), each consisting of eight positions. As shown in Figure 8A, the flow diagram shows that the addition of the X1 and X2 valves could support 19 constructs through 19 sample positions (S1–S19). The sample value (V9H-S) in AKTA Pure^TM^ could provide six positions for sample loading (i.e., S1–S6), one position for PBS (#8), and one (#7) as a connection to V9H-X1 (X1). Similarly, position 8 at X1 was used as a connection to X2, and, therefore, seven positions could be utilized for sample loading (S7–S13). In X2, six more positions were used for sample loading (S14–S19), and two other positions were used for PBS (#7) and sodium hydroxide (#8) to the CIP system-shared-sample lines, columns, and OUTLET fraction collector after each round of sample loading. All the sample loadings were driven by the sample pump and went through a V9 injection valve into a buffer exchange column (BX) connected to column valve V9H-C. A UV detector (UV), a conductivity monitor (Cond.), and a fraction collector were connected through the outlet valve, V9H-O. System PumpA could be utilized for PBS drawing and washing.

As shown in Figure 8C, the antibody protein peak was sufficiently separated from the glycine peak. As shown in Figure 8D, when 19 samples containing identical amounts of 0.5 mg, 1 mg, or 2.5 mg of antibody protein, respectively, were buffer-exchanged with this X1–X2 system, the recovery efficiencies were increased from 35% at 0.5 mg to 75% at 1 mg or 2.5 mg (Figure 8D). As shown in Figure 8E, antibody samples containing 5 mg, 10 mg, 20 mg, 30 mg, 40 mg, or 50 mg could also be buffer-exchanged efficiently, ranging from a recovery rate of 73% to 89% with a similar aSEC profiling (Figure 8F), compatible with the range of protein yields shown in Figure 7. The exchange efficiencies among these protein samples were not statistically different (*p* = 0.79). The results also demonstrate the feasibility of this new system.

## 4. Discussion

**Figure 9:**
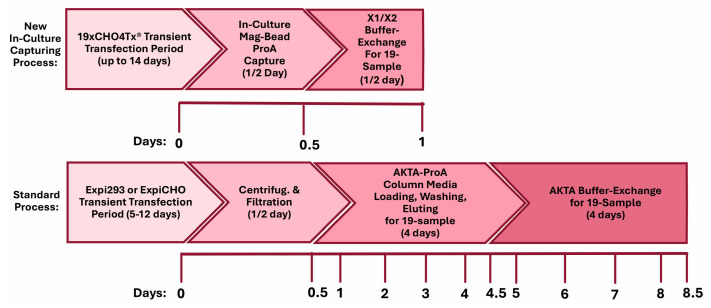
Proposed timelines and a workflow comparison between the new in-culture capturing process and the standard process. For the new in-culture capturing process, nineteen protein eluates from a GenScript SA Plus system (1 h runtime for a maximal 12 samples) can be completely buffer-exchanged by one AKTA X1/X2 system (10 h runtime for a maximal 19 samples) in a half-day. For the standard process, it takes one half-day to generate nineteen 0.2 µ filtered CMs through centrifugation and filtration. It takes four days for one regular AKTA-ProA system (5 samples per AKTA-ProA) to generate nineteen ProA eluates. For the buffer exchange step, it takes one regular AKTA system four days to complete nineteen samples.

Tremendous advances in antibody discovery technologies, along with de novo protein design through machine learning and artificial intelligence, have created hundreds of thousands of antibody candidates available for therapeutic development. These new breakthroughs impose a new challenge for the fast generation of sufficient amounts of antibody proteins for screening and identifying lead candidates. Scalable and consistent TGE technologies with mammalian cells are suitable methodologies for these tasks. TGE-derived therapeutic proteins have a quality on par with that of those produced from stable production and can contribute to faster, more cost-effective drug substance development and manufacturing [28,40]. We have established a new workflow for a robust in-culture antibody-capturing semi-automated production process by employing an innovative and simplified high-titer transient CHO platform. A number of therapeutic antibody candidates against pre-identified disease targets for disease indications of autoimmune, metabolic diseases, and cancers have been produced by this workflow. This process was applied weekly to support the production of dozens of antibodies with sufficient quality and quantity. Figure 9 shows the proposed timelines and a direct comparison between the new in-culture capturing process and the current standard process. After the completion of transient production processes with either Expi293F or ExpiCHO-S, the conventional AKTA-ProA-based purification for nineteen 100 mL productions involves a half-day of harvesting (cell-pelleting centrifugation/medium filtration) and four full days of medium loading into the ProA column, buffer washing, and protein elution with one AKTA-ProA instrument, whereas the magnetic bead in-culture process can be completed in a half-day. The buffer exchange process for the current standard workflow requires four full days with one regular AKTA system, while one X1/X2 system can have 19 samples completed in a single run. This newly proposed workflow should not only shorten the purification timeline but also provide a significant saving on costly filters, centrifugation bottles, and buffers.

The CHO4Tx^®^ transient system reported in this study is a new development for TGE. It does not contain the typical and separate step of DNA complexation with a transfection reagent, nor does it require intermediate feedings. As informed by the provider, CHO4Tx^®^ cells were isolated through “Darwinian” phenotype selection after years of subcultivation. The parental CHOExpress^®^ cell line, a non-cloned clinical manufacturing cell host, is dedicated to stable cell line production. Thus, utilizing CHO4Tx^®^ allows for a smooth transition between the stages of discovery and the preclinical and clinical phases. In comparison with other commercially available transient systems, the CHO4Tx^®^ system consistently achieves high expression titers, even for historically low expressors for the Expi293F™ and ExpiCHO-S™ systems.

Despite the advantages mentioned above for CHO4Tx^®^, there is room for further improvement. One process deficiency of the CHO4Tx^®^ system is the use of two centrifugation steps on the day prior to transfection and on the transfection day. This can represent an obstacle for larger-scale production (>20 L). Efforts are ongoing to reduce or eliminate the centrifugation steps. Since the expression titers for CHO4Tx^®^ are high enough (200 mg/L–500 mg/L), smaller production scales can provide substantial protein materials. Another drawback of CHO4Tx^®^ is the high DNA usage for transfection in comparison with other systems (6.5 mg/L vs. 1 mg/L for Expi293F™ and ExpiCHO-S™). The higher DNA usage is, in part, due to the higher cell density at transfection (12 million cells/mL vs. 3 million cells/mL for Expi293F™ and 6 million cells/mL for ExpiCHO-S™). With a similar transfection efficiency across these host systems, more transfected cells and more transfected DNAs inside cells could result in higher protein production titers. The third disadvantage of the CHO4Tx^®^ system is the 14-day process timeline versus the 5-day process for Expi293F™ and the 7-to-12-day process for ExpiCHO-S™. As shown in Figure 3, the expression titers reached a plateau on day 11, which correlated well with the sharp drop in the VCD beyond days 8–11. This finding suggests that the production process could be shortened from 14 days to 11 days. However, the current 14-day process has the advantage of starting transfection on every workday each week except Monday. In the future, a balance between timeline and process practice feasibility should be considered.

To our best knowledge, this study is the first report detailing in-culture capturing and elution with magnetic ProA beads for transient production. Applying magnetic beads for capturing and separation has long been used for small-scale analysis of nucleic acids and cells [41,42]. Magnetic ProA beads have recently been applied to stable CHO clinical manufacturing for therapeutic antibody production [43,44,45]. The method has been shown to reduce costs, resource utilization, and processing times for protein production. The process also decreases the host cell protein content by reducing cell lysis, and it prevents potential protein loss by improving antibody-capturing efficiency. We have shown that this magnetic bead application can dramatically facilitate the production process for TGE technologies.

This study demonstrated that magnetic bead in-culture protein binding is fast and efficient, whereas the elution process is pH-dependent. Mechanisms for magnetic separation are shown to be similar to those for stirred-tank bioreactors (STRs) in which the binding kinetics depend on the antibody adsorption on the beads and the mixing in the STR [41,42]. The capturing process is, therefore, efficient and not detrimental to cells. One interesting finding is that the in-culture ProA capturing was fully saturated in 30 min, as shown by the pH 3.0 elution, yet it required overnight incubation for a full elution at pH 3.5. This could possibly be due to a higher final pH because of the residual PBS buffer after the bead wash and the buffer effect from the bound antibody protein. An overnight incubation could have potentially weakened the antibody binding to the ProA resin, such that the protein was ready to be eluted at a pH higher than 3.5. The machine-based elution with magnetic beads was found to be similar to that with manual elution. The semi-automation with the GenScript SA plus system, in the capacity of twelve samples at a time, made the repetitive steps of magnetic bead elutions more accurate and reliable.

To increase throughput and capacity, developing automated purification processes is highly desirable. In this study, we installed two additional versatile valves into an AKTA Pure^TM^ system. The combination of the V9H-X1 and V9H-X2 and scouting runs on the UNICORN™ control software enabled up to 19 unique sample injections for the buffer exchange of ProA-purified antibody proteins. A column and system CIP was introduced to avoid any potential cross-contamination among samples. The process is efficient as it can buffer exchange protein samples of as little as 0.5 mg and up to 50 mg with a similar yield and purity, compatible with the production range we observed with the CHO4Tx^®^ host. This automated strategy can be adapted to the step of size-exclusion chromatography and further enhance the protein-polishing capability.

In summary, this study tackled the production challenges of increased numbers of antibody hits by combining a newly developed transient CHO system with a magnetic bead in-culture purification workflow. By pairing X1–X2 AKTA Pure^TM^ automation with a capacity of processing nineteen samples, this workflow could also offer more purification flexibility and significantly shorten the delivery timelines in comparison with other customized preparative purification automation streams [31,35,36,46,47]. The components of the semi-automated workflow have been shown to be efficient and robust, with a capacity of delivering 10 mg of protein. The findings from this investigation should provide a solid foundation for future optimizations in mid-scale automated high-throughput production.

## 5. Conclusions

This study developed a new and robust protein production platform. With the feasibility of its components demonstrated, this new workflow could support the production of dozens of antibodies in sufficient quality and quantity for cell-based assays and biophysical characterization.

## Data Availability

Data is contained within this article.
